# Robust Pressure Sensor in SOI Technology with Butterfly Wiring for Airfoil Integration

**DOI:** 10.3390/s21186140

**Published:** 2021-09-13

**Authors:** Jan Niklas Haus, Martin Schwerter, Michael Schneider, Marcel Gäding, Monika Leester-Schädel, Ulrich Schmid, Andreas Dietzel

**Affiliations:** 1 Institute of Microtechnology, TU Braunschweig, 38124 Brunswick, Germany; m.schwerter@tu-bs.de (M.S.); m.gaeding@tu-braunschweig.de (M.G.); m.leester@tu-braunschweig.de (M.L.-S.); a.dietzel@tu-braunschweig.de (A.D.); 2 Institute of Sensor and Actuator Systems, TU Wien, 1040 Vienna, Austria; michael.schneider@tuwien.ac.at (M.S.); ulrich.e366.schmid@tuwien.ac.at (U.S.)

**Keywords:** pressure sensor, active high-lift, micro-electro-mechanical systems (MEMS), silicon on insulator (SOI), system-in-foil-integration, protective coatings, harsh environment, through glass via (TGV), butterfly wiring, chip-scale package (CSP)

## Abstract

Current research in the field of aviation considers actively controlled high-lift structures for future civil airplanes. Therefore, pressure data must be acquired from the airfoil surface without influencing the flow due to sensor application. For experiments in the wind and water tunnel, as well as for the actual application, the requirements for the quality of the airfoil surface are demanding. Consequently, a new class of sensors is required, which can be flush-integrated into the airfoil surface, may be used under wet conditions—even under water—and should withstand the harsh environment of a high-lift scenario. A new miniature silicon on insulator (SOI)-based MEMS pressure sensor, which allows integration into airfoils in a flip-chip configuration, is presented. An internal, highly doped silicon wiring with “butterfly” geometry combined with through glass via (TGV) technology enables a watertight and application-suitable chip-scale-package (CSP). The chips were produced by reliable batch microfabrication including femtosecond laser processes at the wafer-level. Sensor characterization demonstrates a high resolution of 38 mVV^−1^ bar^−1^. The stepless ultra-smooth and electrically passivated sensor surface can be coated with thin surface protection layers to further enhance robustness against harsh environments. Accordingly, protective coatings of amorphous hydrogenated silicon nitride (a-SiN:H) and amorphous hydrogenated silicon carbide (a-SiC:H) were investigated in experiments simulating environments with high-velocity impacting particles. Topographic damage quantification demonstrates the superior robustness of a-SiC:H coatings and validates their applicability to future sensors.

## 1. Introduction

**Figure 1:**
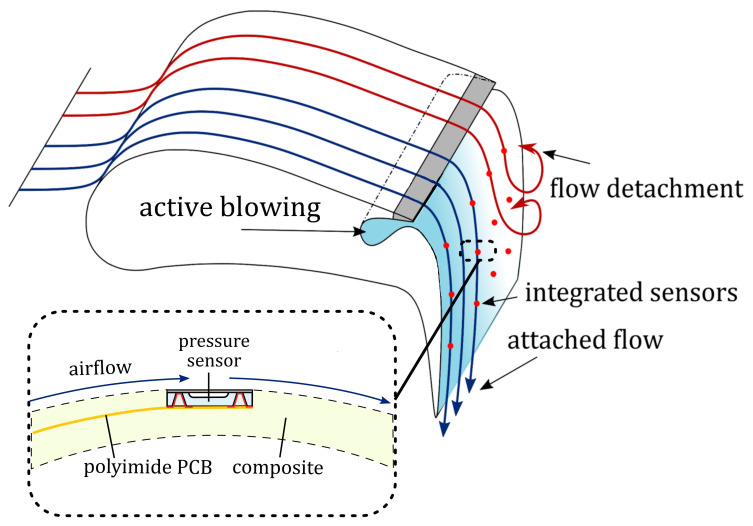
Schematic illustration of an actively controlled high-lift system, utilizing structure-integrated pressure sensors and active blowing (blue) which leads to an attached flow due to the Coanda effect. [2]. The magnified cross-section illustrates the stepless sensor integration into the the Coanda flap.

Traffic concepts of the future will increasingly rely on city airports. The demand for civil aviation in urban areas stands in conflict with the desire for comfortable living with low noise pollution. With slow and steep landing- and take-off trajectories, the area affected by deep overflight can be minimized, allowing for runway lengths of only 800 m [1]. Serving this purpose, actively controlled high-lift systems are being researched, which are based on the principle of active blowing and taking advantage of the Coanda effect [2]. These require precise pressure measurements at places distributed over the airfoil (Figure 1). This is a demanding task, especially in the airfoil regions with a high curvature, since the condition of the flow is very sensitive to surface quality. The smallest protruding structures resulting from surface mounted sensors would negatively influence the flow profile or distort the pressure distribution. Remote pressure sensors [3] rely on small drill holes and can reduce this problem, however, the drill holes on the other hand add some roughness to the surface and the tubing delays the pressure recordings. To address this problem, flush integrable MEMS pressure sensors have been investigated, which minimize the flow distortion at the location of interest [4], but still make use of bond wires to connect to the chip. Although silicon vias have been shown to eliminate wire bonds [5], fragile and active sensor elements on the sensor’s exterior still restrict their utilization to laboratory conditions. The authors have previously presented a surface flush pressure sensor with active elements enclosed, making it suitable for the harsh environment of a water tunnel and real-world applications [6,7,8]. This sensor design, however, utilizes adhesive bonding, which cannot be considered as a hermetic enclosure of the reference pressure volume. Furthermore, the utilization of an adhesive limits the sensor’s temperature range, consequently excluding soldering techniques for substrate bonding. To eliminate these disadvantages, a new sensor design is presented herein. This surface-integrated pressure sensor could also be of high interest for various other aerodynamic measurement tasks, such as in wind turbines.

## 2. Sensor Concept

The flush integrable MEMS pressure sensor with an ultra-smooth surface was developed to be highly sensitive and long-term stable and not to influence the flow or to delay the recording of pressure present on the airfoil’s surface (Figure 1). Small lateral sensor dimensions shall allow a seamless integration of the rigid sensor into the surface of curved structures while a thickness of only 200 μm will occupy only one composite layer, which can be locally left out during airfoil production. Furthermore, the integration of such ultra-thin chips in flexible foils with interconnecting and in-foil wiring has been investigated in earlier works [9,10,11,12].

As in previous sensor designs, any electrically active paths need to be enclosed inside the sensor body to guarantee robustness as well as water resistance.

After the complete removal of the SOI handle layer (see Section 3.6 for details), the thin remaining device layer will form the sensor membrane. Its silicon oxide-covered surface can be directly exposed to the harsh environment of a wind- or water tunnel or any environment an aircraft can experience. Protective coatings of silicon-nitride and silicon carbide (presented in Section 4) can complement the surface passive sensor design to provide more robustness against abrasion by particle impact.

**Figure 2:**
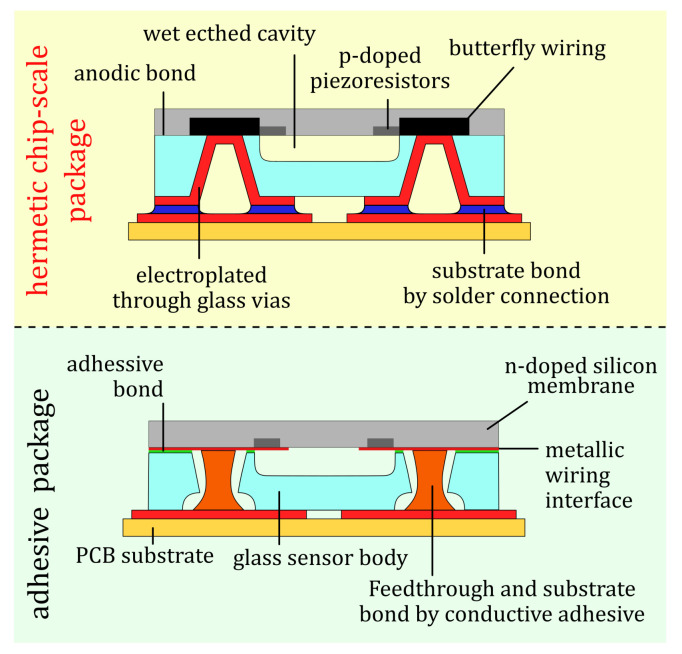
Schematic cross-sections of the hermetic CSP (**top**) in comparison with the earlier adhesive package sensor concept [6,7,8] (**bottom**).

**Figure 3:**
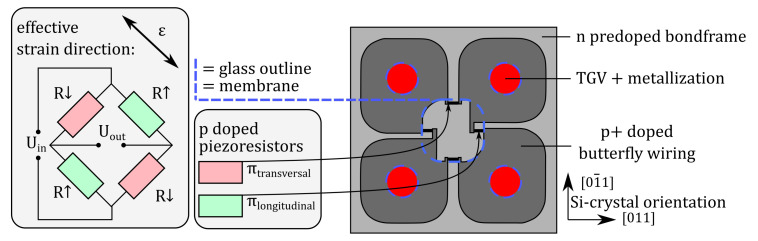
Schematic illustration of the Wheatstone circuit implementation (**left**) and the butterfly wiring scheme (**right**).

To reduce manufacturing tolerances, as well as to improve the applicability of the sensor, several changes were made compared to earlier pressure sensors (cf. Figure 2): a sensor layout, which replaces metal feedthroughs by highly doped silicon feedthroughs in butterfly geometry (Figure 3) allows for hermetic enclosure by anodic bonding. The metallization of fs-laser-made TGV sidewalls replaces manually dispensed conductive adhesives in the via. These TGVs establish an electrical connection to the silicon feedthroughs and simultaneously allow a CSP with contacts on the sensor’s bottom side. Furthermore, this concept allows uninterrupted wafer-level fabrication. Since these changes eliminate the need for using any adhesives, which previously limited the suitable temperature range, the presented sensor chips can be handled and soldered with state-of-the-art substrate bonding techniques.

The introduction of SOI wafer technology grants an ultra-smooth sensor surface and perfectly predictable and homogeneous membrane thickness over all sensors.

## 3. Sensor Elements and Their Microfabrication

**Figure 4:**
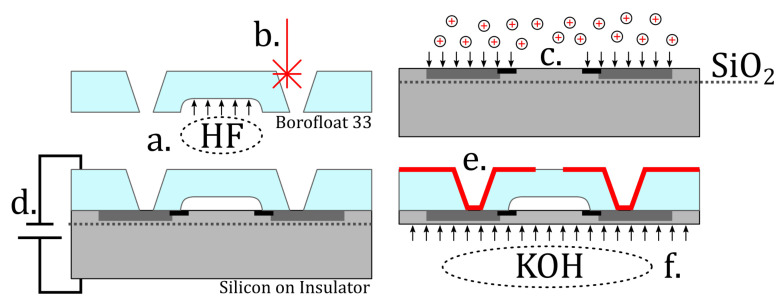
Schematic illustrating the production steps: (**a**) pressure reference cavity by HF wet etching into a 200 μm Borofloat glass wafer; (**b**) TGV holes by femtosecond laser ablation; (**c**) the two-step local boron doping of piezoresistors and butterfly wiring; (**d**) anodic bonding of silicon and glass wafers; (**e**) metallization and realization of a CSP by gold magnetron sputtering and copper electroplating; and (**f**) membrane thinning by KOH wet etching, where the SOI silicon oxide layer serves as reliable etch stop layer.

Figure 4 illustrates the main fabrication steps (a)–(f), which are described in detail in the following subsections.

### 3.1. Pressure Reference Cavity Etching (a)

For each sensor, a nitrogen-filled reference atmosphere with a pressure of 1 (at 20 ${}^{\circ }C$) was encapsulated in the glass cavity by a deformable silicon membrane. As a first production step, the cavities were etched into 200 μm floated borosilicate glass wafers (Borofloat 33, Schott AG, Mainz, Germany). After passivation with a magnetron sputtered and subsequently photographically structured gold hard mask layer, the glass was etched in a solution of deionized water, phosphoric acid and hydrofluoric acid (48.57% H${}_{3}$PO${}_{4}$ + 44.57% H${}_{2}$O + 6.86% HF). Increasing the reference volume also increases the linearity of the deflection-mode sensor, but the alignment with the piezoresistors becomes more difficult to control. Because the wet etching of glass is an isotropic process, the etched depth was also etched in the lateral dimension, which has to be taken into account for the proper positioning of the piezoresistors. Since an opaque hard mask is used, the actual etching progress cannot be directly observed, so that only the etched depth indicates the lateral extension of the cavity. The process is terminated, after a cavity depth of 35μm has been reached, which results in a lateral cavity dimension of 600 (=530 + 2 × 35) μm.

### 3.2. Through Glass Holes by Femtosecond-Laser Ablation (b)

**Figure 5:**
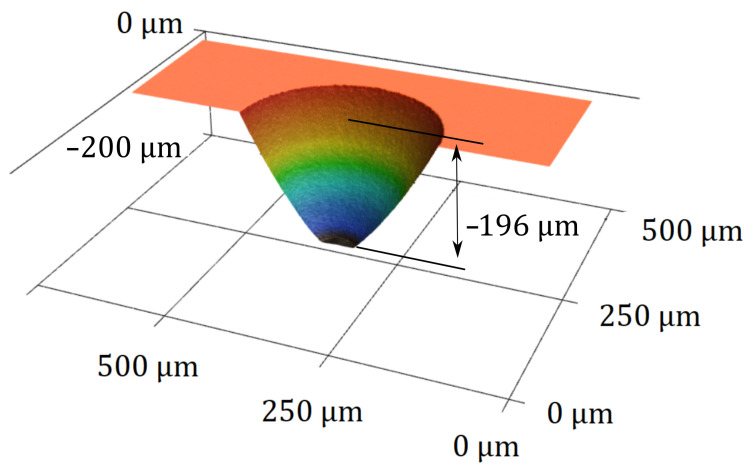
3D profile of a through hole produced by means of femtosecond laser ablation and consecutive smoothing obtained with a laser scanning microscope (VK-X260K, Keyence, Osaka, Japan).

Metallized through glass holes, referred to as TGVs, facilitate an electrical connection from the sensor periphery to the later silicon interface. To create the through glass holes, a laser micromachining workstation (microSTRUCT C, 3D Micromac, Chemnitz, Germany), equipped with a (Yb:KGW) femtosecond laser source (PHAROS, Light Conversion, Vilnius, Lithuania) was used, which emits at λ= 1028 nm. For fast patterning, the laser beam was deflected using a laser scanner (Intelliscan 14, Scanlab GmbH, Puchheim, Germany) and a f=100 mm telecentric FTheta Lens (Linos AG, Göttingen, Germany). The pulse energy (cf. Table 1) for the structuring process was selected not too far above the ablation threshold of 8,99 μJ for borosilicate glass [6]. Laser ablation using higher-energy pulses removes material faster, but causes increasing particle re-depositioning and glass corrosion in the adjacent regions. The tapered through glass holes exhibit a max. diameter of ${⌀}_{max}$ = 300 μm (${⌀}_{min}$≈ 69 μm, α = 30, cf. Figure 5). This hole geometry allows continuous via metallization by magnetron sputtering, free of any interruptions. Finally, any redeposited glass particles are removed and rough edges are smoothened by a 30 s dip in a glass etching solution described in Section 3.1. Figure 5 shows the finally obtained topography of a through glass hole.

### 3.3. Piezoresistor Fabrication and Butterfly Wiring (c)

The sensor’s pressure-dependent membrane deflection is evaluated using piezoresistive silicon elements, which are aligned with the edges of the glass cavity. A Wheatstone circuit was realized within the device layer by boron doping. Different doping levels are used to create piezoresistors with high piezoresistive coefficients, and large-area p+ doped conductive paths, which interconnect the Wheatstone bridge, replacing the former metal wiring (cf. Figure 2). Due to its shape, it is referred to as butterfly wiring. The piezoresistors have a geometry of 100×10$\mu m^{2}$ and a boron doping concentration of $N_{a}\approx 5\times 10^{19}/cm^{-3}$, resulting in a resistance of approx. 1 kΩ and high piezoresistive coefficients (cf. Table 2). The conductive butterfly wiring is highly boron doped ($N_{a}\approx 5\times 10^{20}/cm^{-3}$), which leads to a specific electrical resistance of approx. 2 mΩ · cm and yields a parasitic resistance of approx. 17 Ω between adjacent piezoresistors. In order to affect the sensor output only minimally, the wiring is mostly located outside those areas, which experience high mechanical stress due to membrane deflection:

(1) $$\pi_{longitudinal}=\frac{1}{2}\left(\pi_{11}+\pi_{12}+\pi_{44}\right)$$

(2) $$\pi_{transversal}=\frac{1}{2}\left(\pi_{11}+\pi_{12}-\pi_{44}\right)$$

For production of the membrane, the device layer of an SOI (see Table 3 for details) wafer is processed: the thermal oxidation of the wafer is performed using an LPCVD diffusion furnace (Centronic E1200 HT260-4, Centrotherm; Blaubeuren, Germany) for 30 min at 1100 ${}^{\circ }C$. In those regions, where dopants shall be introduced into the silicon lattice, the previously applied photoresist (Ma-P 1215, Micro resist technology, Berlin, Germany) is lithographically removed and the oxide is consecutively structured using 40% hydrofluoric acid (HF). After stripping the photoresist, 500 μL boron formulation (Borofilm 100, Emulsitone Chemicals, Washington, DC, USA) is spin-coated onto the wafer, cured (hotplate, 20 min @ 200 ${}^{\circ }C$), and thermally driven into the silicon (LPCVD furnace, 30 min @ 900 ${}^{\circ }C$), forming four piezoresistors. In a second step, the butterfly wiring is similarly produced, but in a higher diffusion temperature (LPCVD furnace, 30 min @ 1100 ${}^{\circ }C$). After each diffusion, the masking oxide is removed by a dip into 40% HF. Figure 3 illustrates the piezoresistors and the butterfly wiring.

### 3.4. Hermetic Sealing by Anodic Wafer Bonding (d)

**Figure 6:**
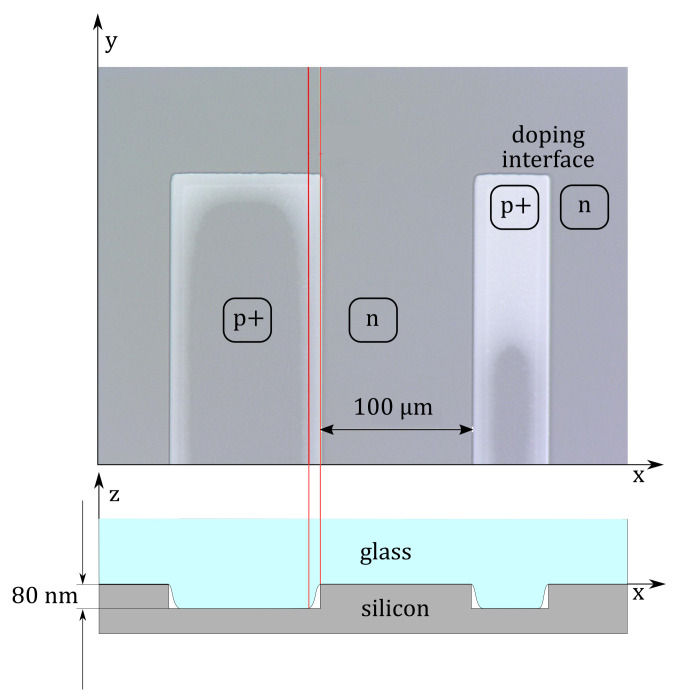
Top-view micrograph of a test structure with differently doped silicon regions, anodically bonded to glass. Lighter gray areas reveal the formation of non-bonded channels along the interface between differently doped silicon areas. A schematic cross-section illustrates this problem.

A permanent hermetic enclosure of the reference atmosphere is crucial for reliable pressure sensing. Anodic bonding is known to create a hermetic seal between silicon and glass [13,14]. To achieve a planar silicon-to-glass interface, the former metal wiring interconnecting the doped piezoresistors is replaced by a highly boron-doped silicon butterfly wiring (cf. Figure 3). Despite the hermetic properties of the anodic bond, it was found that only with a doping-wise uninterrupted bond frame (as presented in Figure 3) can a hermetic seal be achieved. Long-term gas exchange between the reference cavity and the surrounding atmosphere shall not occur even in the presence of a 80 nm step between neighboring but differently doped silicon areas and the glass lid. (cf. Figure 6).

For production, the silicon wafer is precisely aligned with the structured glass wafer using a mask aligner (EVG 620, EVG, St. Florian am Inn, Austria), equipped with a special bond tool for handling. At this moment, thin metal spacers “flags” between the wafers allow a gas-exchange with the environment. After inserting the bond tool with the aligned wafers into the anodic bonding chamber (AB-1 PV, EVG, St. Florian am Inn, Austria), the chamber is flushed with nitrogen gas. At room temperature, the nitrogen-filled bond chamber is hermetically sealed. After heating to 400 ${}^{\circ }C$, which also leads to a significant rise in pressure, the flags are extracted, thus the two wafers make contact and the anodic bonding is carried out at 600 V for 30 min. The anode pressure is set to 75 kPa. Due to the enclosed atmosphere, the encapsulated reference pressure returns to the initial pressure, after cooling to room temperature.

It is important to state that the coefficients of the thermal expansion of the utilized glass and silicon must match for successful bonding: $\alpha_{borofloat33}\approx \alpha_{silicon}=\;3.25\times 10^{-6}$${}^{\circ }$C${}^{-1}$ in the range between 20 ${}^{\circ }C$ and 400 ${}^{\circ }C$.

### 3.5. Through Glass via Plating and Terminal Metallization (e)

**Figure 7:**
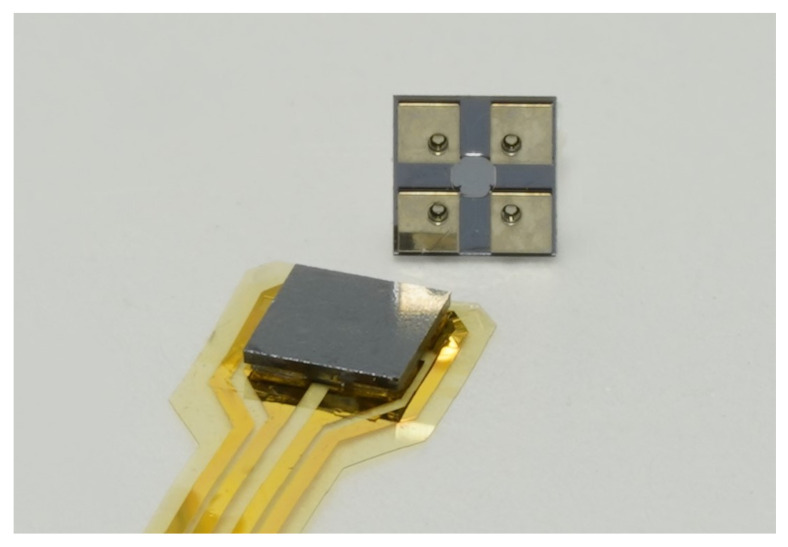
Photography of the sensor, soldered onto polyimide substrate and ready for flush integration into composite material (**left**). Sensor’s bottom view showing through glass vias of the CSP (**right**) with outer dimensions of $2.2\times 2.2\times 0.2\;mm^{3}$.

After wafer bonding, electrical contacts to the butterfly wiring (cf. Figure 2) have to be established. After removing native oxide from the silicon bottom side inside the TGVs by reverse sputtering, a layer of chromium (9 nm) and a layer of gold (300 nm) are sequentially magnetron sputtered (LS 440 S, von Ardenne Anlagentechnik GmbH, Dresden, Germany) onto the entire glass backside as well as the TGV walls and bottom, thereby contacting the butterfly wiring. These layers serve as the start layer for the subsequent copper electroplating, for which the isolation areas between the individual terminals of the CSP are masked with photoresist. After electroplating (15 μm), the resist is stripped and the gold and chromium layers are chemically removed (TechniEtch-AC35 for gold and TechniEtch-Cr01 for chromium, Microchemicals GmbH, Ulm, Germany), thus producing the four 950μm×950μm CSP terminals, as displayed in Figure 7.

### 3.6. Membrane Thinning (f)

The 10 μm-thick device layer of the SOI wafer is released by completely removing the handle layer by wet etching in 40% aqueous KOH solution at 80 ${}^{\circ }C$. After approximately 7 h, the handle layer is completely removed. Finally, the wafer is diced into 24 × 24 chips with a lateral dimension of 2.2×2.2 $mm^{2}$.

## 4. Protective Coating

For the CSP with flip-chip mounting, housing becomes obsolete and the chemically inert sensor membrane surface is directly exposed to harsh environments. In order to provide a more robust protection against impacting particles, thin-film layers of amorphous hydrogenated silicon carbide and silicon nitride are investigated.

### 4.1. Preparation of Protective Coatings

Amorphous hydrogenated silicon nitride (a-SiN:H) and silicon carbide (a-SiC:H) thin films were deposited on double-side polished n-type (phosphorous doped) (100) silicon wafers with a thickness of 360 μm using an inductively coupled plasma-enhanced chemical vapor deposition (ICP-CVD) downstream reactor (PlasmaLab 100, Oxford Instruments, Bristol, UK). The RF plasma source operates at a frequency of 13.56 MHz. Prior to deposition, the chamber was evacuated down to a base pressure of $9\times 10^{-5}$ Pa and heated to the deposition temperature. As precursor gases, N${}_{2}$ and SiH${}_{4}$ were used for a-SiN:H, and CH${}_{4}$ and SiH${}_{4}$ for a-SiC:H with Ar as neutral background gas in both cases. The deposition parameters were detailed in Table 4. The film thickness was determined using spectral reflectance (F20-UVX, Filmetrics, Unterhaching, Germany) and the film stress was measured using capacitive wafer bow gauge (MX203-6-33, E+H Metrology, Karlsruhe, Germany). The parameters for the a-SiC:H thin film were chosen to obtain a high hardness of 23 GPa. Details concerning both thin-film systems have been reported earlier [15,16].

### 4.2. Experimental Setup for Controlled Abrasive Stress

**Figure 8:**
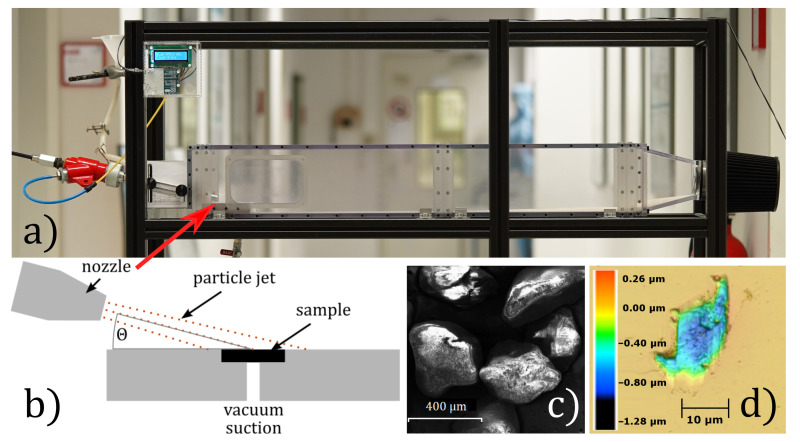
(**a**,**b**) Photograph and schematic of the setup utilized for reproducible particle blasting experiments, (**c**) the electron micrograph of the particles (silica sand), and (**d**) laser scanning microscope (LSM) image of a typical surface damage topography resulting from particle impact.

To determine the robustness of the thin-film coatings, a laboratory setup simulating environmental abrasive damaging in particle-loaded flows was established (cf. Figure 8). Silica sand with a hardness of 1200HV ( 11.77 GPa), an average particle diameter of 350 μm and an apparent density of 1.2 g $cm^{-3}$ was accelerated by an air jet. This jet was directed towards a 2×2 $mm^{2}$ sample, leading to collisions of the particles with the sample surface. Aside from the material properties of the particles and the target surface, the crucial parameters of the particle blasting process were the angle of particle incidence and the particle velocity. A 2-DOF fixture of the jet nozzle allows for the exact adjustment of the blasting distance and incidence angle Θ over a wide range. Utilizing micro controller monitoring, the jet speed is adjusted to 100 m s^−1^. An acrylic housing prevents ambient influences.

Figure 8c shows an electron micrograph of the utilized silica sand. The jet speed is regulated to a constant value of 100 m s^−1^. Θ is set to the values 1 ${}^{\circ }$, 3 ${}^{\circ }$, 6 ${}^{\circ }$, 9 ${}^{\circ }$ and 12 ${}^{\circ }$. Six samples of each combination of coating material and Θ were investigated.

### 4.3. Damage Quantification

A laser scanning microscope (VK-X260K, Keyence, Osaka, Japan) was used to determine the damages on the sample’s surface within a defined area. Three-dimensional images were recorded, allowing for subsequent analysis to digitally determine the dimensions and the topography of damages, as shown in Figure 8d). For allowing statistics, an area of 0.78 mm was analyzed by merging 4×4 single LSM images. Using a 50× objective with a numerical aperture of 0.95 a depth resolution of 0.5 μm could be achieved. To suppress the influence of the roughness, a lower threshold for recess detection was set to 0.035 μm. Each recess defect was automatically evaluated for its volume. As the value representing the damage level, the sum of all damage volumes in the evaluation area was calculated.

## 5. Results and Discussion

A robust pressure sensor was developed to measure static and dynamic pressures at the easily disturbed flow boundary layer of a high-lift system. The use of silicon-on-insulator wafers grants a very well-defined and consistent membrane thickness over all sensors. The CSP does not affect the flow by bond-wires, pressure measuring holes, surface steps, indentations or protrusions. Mounting onto a flexible substrate (e.g., polyimide) is realized by flip-chip bonding. Both can be integrated into the fabrication process of composite structures. Thereby, a fully passivated pressure measuring point flush with the airfoil surface can be created. The CSP is completely fabricated at the wafer level, thereby eliminating manual adhesive bonding steps. Moreover, the implementation of a flat four-terminal no-lead CSP (inspired by a QFN *Quad Flat No-lead* package) facilitates manual or automated chip handling and allows for substrate bonding by conventional soldering techniques, since process temperature limits associated with adhesives are no longer an obstacle. The CSP allows for handling with standard pick and place suction tools without damaging the sensor. Reflow soldering using solder paste, as well as manual soldering using solder wire, was proven to be possible. Figure 7 shows the CSP and the mounting on flexible PI foil by reflow soldering. This type of environmentally passivated pressure sensor promises much better usability and applicability than previous versions.

**Figure 9:**
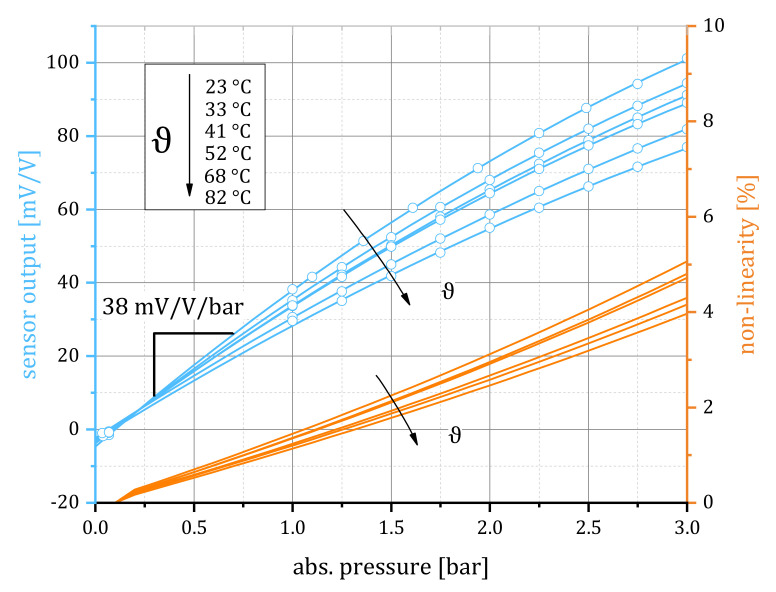
Exemplary pressure sensor calibration curves: sensor output (blue) depending on the ambient temperature. Blue lines represent polynomial (second order) least squares fitting to the data points. Linearity errors (orange) are calculated as the deviation of the polynomial fit from the linear fit in the corresponding interval.

The ratio of the input-to-output sensor voltage $U_{out}/U_{in}$ from the sensor’s Wheatstone circuit (cf. Figure 3) is recorded at varying pressures from 0 to 3 and temperatures from 20 ${}^{\circ }C$ to 80 ${}^{\circ }C$. The resulting calibration curves of an exemplary sensor, as given in Figure 9, show a sensitivity of 38.33mV/V/bar, a moderate temperature dependency, and a linearity error ≤3% for pressures not exceeding 2.1 at 20 ${}^{\circ }C$. For higher and for lower temperatures, the sensor behavior can be extrapolated assuming a linear response to temperature.

**Figure 10:**
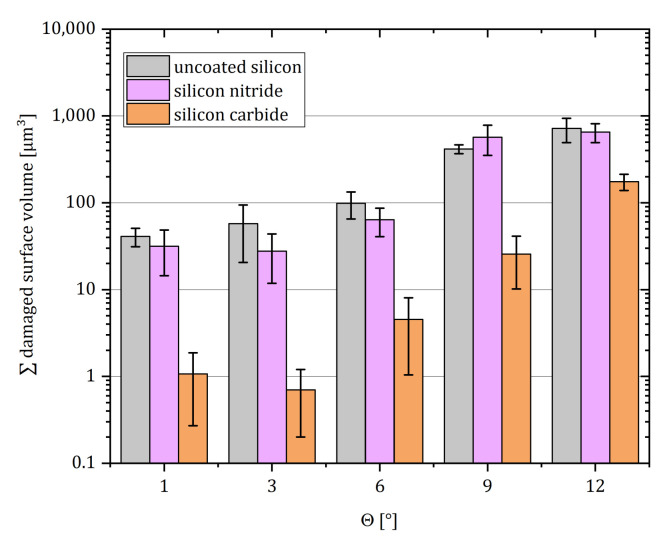
Cumulated surface damage volume in dependence of Θ. Six samples were evaluated for each data point. The error bars represent the standard deviation.

Figure 10 represents the summed damage volume per 0.78 mm^2^ surface in dependence of the incidence angle for samples coated with different protection layers. While for incidence angles $\Theta <9{}^{\circ },$ optical inspections merely show superficial scratches in the coating, leaving the silicon undamaged, abrasion angles of $\Theta \geq 9^{\circ }$ create voids in the bulk material for all samples. While coating with silicon nitride did not show significant protection, silicon carbide thin films significantly reduce the damage by approximately 80% for all tested Θ. These thin-films can in the future be directly deposited onto the sensor surface at the wafer-level following the removal of the handle layer (cf. Section 3.6) in order to protect the membranes against impacting particles in harsh environments without adding noteworthy thickness to the membrane.

## 6. Conclusions and Outlook

In this work, a robust MEMS pressure sensor for use in harsh wind and water tunnel experiments as well as in real aircrafts was developed. SOI wafer technology allows for an exact, reproducible and homogeneous thickness of the membrane. Anodic wafer bonding is enabled by the new sensor-internal butterfly wiring concept based on highly doped large-area interconnections between adjacent piezoresistors. These replace the former metal wiring, which would make anodic bonding impossible due to surface steps. The fabrication of a CSP eliminates any manual production steps at the chip level. This drastically increases the production yield per wafer, decreases the effective production time, and also facilitates the option of machine aided chip-handling. Sensor calibrations at different temperatures prove the high sensor resolution of 38 mVV^−1^ bar^−1^ and a linear transfer function. The observed temperature effect can be easily digitally compensated using external temperature measurements. At temperatures below 0 ${}^{\circ }C$, this observed trend will certainly continue in principle. However, it would be interesting to investigate the effect of icing and deicing on the sensor behavior in the future.

The thin sensor chip, mounted on a flexible polyimide foil, may in the future be combined with previously developed hot-film sensors [17] for combined pressure and wall shear stress determination which is essential for active flow control. A possible monolithic combination of pressure- and hot-film sensors utilizing a heated sensor membrane could also contribute to deicing approaches.

A special sand-blasting setup was established to test thin-film coatings of silicon nitride and silicon carbide for their protective capabilities against abrasive damaging within harsh environments. Silicon carbide coating, which offers good protection in the future can be directly applied to the sensor surface exposed to the measuring environment to make the sensor even more robust. 

## Figures and Tables

**Table 1:** Laser fabrication parameters for the through glass holes.

Parameter	Value
Wave length	1028nm
Repetitions	60
Pulse energy	10.36 μ J
Pulse duration	236fs
Scan speed	500 mm s^−1^
Repetition rate	100kHz
Spot diameter	25μm
Filling pattern	conc. circles, d=2.5μm

**Table 2:** Piezoresistive coefficients π in [$10^{-11}/\mathrm{Pa}$^−1^] of the piezoresistors at 25 ${}^{\circ }C$. $\pi_{longitudinal}$ and $\pi_{transversal}$ are calculated for the <100> crystal direction according to Equations (1) and (2).

$\pi_{11}$	$\pi_{12}$	$\pi_{44}$	$\pi_{\mathrm{longitudinal}}$	$\pi_{\mathrm{transversal}}$
1.98	−0.33	41.43	21.54	−19.89

**Table 3:** SOI wafer specifications (Vendor: Si-Mat, Silicon Materials).

Feature	Device Layer	Oxide Layer	Handle Layer
Type/orientation	N/Ph <100> 0.5${}^{\circ }$	-	N/Ph <100> 0.5${}^{\circ }$
Thickness	10±0.5 μ m	1±0.01 μ m	300±15 μ m
Resistivity	1–10 Ω cm	-	1–10 Ω cm
Finish	Polished	-	Polished

**Table 4:** Deposition and film parameters of a-SiC:H and a-SiN:H.

Deposition Parameter	a-SiC:H	a-SiN:H
CH${}_{4}$ flow (sccm)	13.5	0
SiH${}_{4}$ flow (sccm)	6.5	12
N${}_{2}$ flow (sccm)	0	10
Ar flow (sccm)	50	48
Power (W)	2000	750
Deposition temperature (${}^{\circ }$C)	250	350
Deposition pressure (mTorr)	10	7
Film thickness (nm)	311	320
Film stress (MPa)	−653	269

## Data Availability

The raw data of the experiments can be requested from the authors.
